# Antithrombotic and antiplatelet activities of Soshiho-tang extract

**DOI:** 10.1186/1472-6882-13-137

**Published:** 2013-06-18

**Authors:** Jung-Jin Lee, Taesoo Kim, Won-Kyung Cho, Jin Yeul Ma

**Affiliations:** 1 Korean Medicine (KM)-Based Herbal Drug Research Group, Korea Institute of Oriental Medicine, 1672 Yuseong-daero, Yuseong-gu, Daejeon 305-811, Republic of Korea

**Keywords:** Soshiho-tang, Antithrombotic activity, Antiplatelet activity, Serotonin secretion, TXB_2_ formation

## Abstract

**Background:**

Soshiho-tang (SH; Chinese name, Xiao-Chai-Hu-Tang; Japanese name, Shosaiko-to) is a traditional Korean, Chinese, and Japanese medicine, which has been used to treat various conditions, including hepatitis, liver cirrhosis, and chronic and acute liver disease. SH consists of seven herbal components, of which *Scutellaria baicalensis* Georgi and *Zingiber officinale* Roscoe, are reported to have antithrombotic and antiplatelet activities. We investigated the antithrombotic activity of SH, including *S. baicalensis* and *Z. officinale*, as an integrative therapy.

**Methods:**

To identify the antithrombotic activity of SH, we used a FeCl_3_-induced thrombus formation model. The mechanism of SH-mediated antithrombotic activity was assessed by determining platelet aggregation and coagulation times *ex vivo*, washed platelet aggregation, serotonin secretion, and thromboxane B_2_ formation.

**Results:**

SH prolonged the occlusion time of thrombus formation when applied in a FeCl_3_-induced thrombus formation model. SH also inhibited collagen-induced platelet aggregation *ex vivo* in a concentration-dependent manner; however, it did not affect coagulation. Hence, to identify the antiplatelet effect of SH, we investigated washed platelet aggregations *in vitro*. SH significantly inhibited various agonist-induced platelet aggregations, and it completely inhibited serotonin secretion and thromboxane B_2_ formation.

**Conclusions:**

The findings suggest that SH inhibited FeCl_3_-induced thrombus formation through antiplatelet activity, including inhibition of platelet aggregation, and serotonin and TXB_2_ production. Thus, SH may be useful as an integrative herbal formula for the treatment of thrombosis.

## Background

Thrombus formation is a pivotal event in the pathogenesis of cardiovascular disease. Platelets are critical in all phases of thrombus formation, including the initial steps of atherosclerosis, progression of fatty streaks to atherosclerotic lesions, and any resulting thrombotic complications [1]. Presently, thrombotic disease is clinically treated by the administration of acetylsalicylic acid (aspirin), adenosine diphosphate (ADP) receptor blockers, and glycoprotein (GP) IIb/IIIa antagonists as directed to prevent cardiovascular disease [2]; however, they can have systemic hemorrhagic side effects [3]. Thrombus formation is also directly connected to the initiation of coagulation in fibrin production. Blood coagulation factor VIIa (FVIIa) is important in tissue factor complex formation [4]. Following tissue injury, membrane-bound tissue factor (TF) crucially binds to FVIIa and the binary FVIIa/TF complex (the extrinsic pathway), then generates FIXa (the intrinsic pathway) and FXa, which result in formation of the Xnase complex [5-7]. The Xnase complex, along with FVIIa/TF, converts FX to activated factor X, which assembles with activated factor V to form the prothrombinase complex that is directly responsible for the formation of thrombin [7].

In the atherosclerotic artery, platelets are activated to respond to injury by plaque rupture or erosion, which leads to the release of additional agonists, such as thromboxane (TX) A_2_, 5-hydroxytryptamine (serotonin), and ADP, which lead to further recruitment of platelets to injury sites [8-10]. TXA_2_ has several effects as it is the major contributor of platelet aggregation via collagen, which is the most atherogenic activator of the vessel wall [11]. Meanwhile, serotonin is released from dense granules during platelet activation. In a previous report, serotonin was shown to play a role in platelet formation, and was related to the constriction and dilation of vascular vessels [12].

Soshiho-tang (SH) is a traditional Korean, Chinese, and Japanese medicine, and has been used to treat various conditions, including hepatitis, liver cirrhosis, and chronic and acute liver disease [13,14]. Recent studies show that SH has various pharmacological properties, including immunomodulatory effects [15], modulation of liver fibrosis [16], and treatment of anti-interstitial pneumonia [13,14]. However, the integrative effect of SH on antithrombotic and antiplatelet activity have not reported with regard to its seven herbal components, including *Bupleurum falcatum* Linne, *Glycyrrhiza uralensis* Fischer, *Panax ginseng* C.A. Meyer, *Pinellia ternata* Breitenbath, *Scutellaria baicalensis* Georgi, *Zingiber officinale* Roscoe, and *Zizyphus jujuba* Miller var. *inermis* Rehder. Among these components, *S. baicalensis* and *Z. officinale* have been reported to have antithrombotic and antiplatelet activity [17-19]. Hence, in this study, we investigated the antithrombotic activity through the antiplatelet effects of SH including the use of *S. baicalensis* and *Z. officinale* as integrative therapies.

## Methods

Indomethacin, bovine serum albumin (BSA), ethylene glycol-bis(*β*-aminoethyl ether)-N,N,N',N'-tetraacetic acid (EGTA), serotonin creatinine sulfate, *o*-phthalaldehyde (OPT), imipramine, acetylsalicylic acid (ASA, aspirin), and dimethyl sulfoxide (DMSO) were obtained from Sigma Chemical Co. (St. Louis, MO, USA). Collagen, arachidonic acid (AA), ADP, and thrombin were purchased from Chrono-Log Co. (Havertown, PA, USA). Cephalin, thromboplastin, and bovine thrombin were purchased from Instrumentation Laboratory Co. (Milano, Italy). Other chemicals were of analytical grade.

### Animals

Male Sprague–Dawley rats (250–300 g) and New Zealand white rabbits (2.5-3 kg) were purchased from Sam-Tako Animal Co. (Osan, Korea) and acclimated for 1 week at a temperature of 24 ± 1°C and humidity of 55 ± 5%. The animals had free access to a commercial pellet diet obtained from Samyang Co. (Wonju, Korea) and drinking water. The animal studies have been carried out in accordance with the Korea Institute of Oriental Medicine Care Committee Guidelines, and were approved by the Korea Institute of Oriental Medicine Care and Use Committee (Protocol # 12–056). The animals were cared for in accordance with the dictates of the National Animal Welfare Law of Korea.

### Preparation of Soshiho-tang extract

Bupleurum Root, Glycyrrhizae Radix et Rhizoma, Ginseng Radix, Pinellia Tuber, Scutellaria Root, Zingiberis Rhizoma Crudus, and Zizyphi Fructus were purchased from Yeongcheon traditional herbal market (Yeongcheon, Korea). All voucher specimens were deposited in the herbal bank of the KM-Based Herbal Drug Research Group, Korea Institute of Oriental Medicine.

SH was prepared according to previously reported methods [16]. Briefly, 1674.5 g of medicinal herbal drug, including Bupleurum Root 600 g, Glycyrrhizae Radix et Rhizoma 100 g, Ginseng Radix 200 g, Pinellia Tuber 200 g, Scutellaria Root 400 g, Zingiberis Rhizoma Crudus 74.5 g, and Zizyphi Fructus 100 g, was decocted with 16.745 L of boiling water in a stainless oven for 3 h at 115°C using a Gyeongseo Extractor Cosmos-600 (Incheon, Korea), after which the decoction was filtered using standard testing sieves (150 μm; Retsch, Haan, Germany). The filtrate was lyophilized and stored in desiccators at 4°C. The freeze-dried extract powder was then dissolved in 50% DMSO (v/v with phosphate-buffered saline) and filtered (pore size, 0.2 μm), then kept at 4°C for use.

### Arterial thrombus formation *in vivo*

Male Sprague–Dawley rats (n = 7) were orally administered with SH (300 and 600 mg/kg) or ASA (100 mg/kg), a positive control, for 5 days, and then anaesthetized by intraperitoneal injection of pentobarbital (50 mg/kg). Arterial thrombus formation *in vivo* was investigated as previously described [20]. Briefly, a segment of the right carotid artery was isolated and dissected free of the vagus nerve and surrounding tissues. Aortic blood flow was measured with a Blood FlowMeter (ADInstruments, Colorado Springs, CO, USA). Arterial thrombus formation was induced by wrapping a 2-mm^2^ Whatman Grade 1 filter paper, saturated with 50% ferric chloride (FeCl_3_; w/v, in distilled water), on the carotid artery near the probe for 10 min. The time needed for occlusion to occur was measured for up to 60 min, and occlusion time was assigned a value of 60 min for vessels that did not occlude within that time.

### Platelet aggregation and coagulation times *ex vivo*

*Ex vivo* platelet aggregation was investigated as previously described [20]. In brief, male Sprague–Dawley rats (n = 8) were orally administered with SH (300 and 600 mg/kg) and ASA (100 mg/kg) for 5 days, and blood was collected 60 min after the last administration. Platelet-rich plasma (PRP) was obtained by centrifuging the blood sample at 180 × *g* for 10 min, and platelet-poor plasma (PPP) was obtained by centrifuging the PRP at 2100 × *g* for 10 min continuously. PRP was adjusted to 4 × 10^8^ platelets/ml with PPP. Platelet aggregation was measured with an aggregometer (Chrono-Log Co.), and collagen (3 μg/ml) and ADP (5 μM) were used as aggregation stimulators. The plasma-activated partial thromboplastin time (APTT) and prothrombin time (PT) were automatically measured with an Automated Coagulation Laboratory 100 Instrument (Instrumentation Laboratory) as previously described [20]. In brief, PPP was incubated at 37°C for 7 min, and then 100 μl incubated plasma was mixed with 50 μl cephalin in the process plate. Coagulation was triggered by the addition of CaCl_2_ plus either 100 μl thromboplastin or 100 μl polibrene for the APTT and PT assays, respectively.

### Washed rabbit platelet preparation and platelet aggregation *in vitro*

Blood was withdrawn from the ear artery of male New Zealand white rabbits and collected into 0.15 (v/v) of anticoagulant citrate dextrose (ACD) solution that contained 0.8% citric acid, 2.2% trisodium citrate, and 2% dextrose (w/v). Washed platelets were prepared as previously described [20]. Briefly, PRP was obtained by centrifugation of rabbit blood at 230 × *g* for 10 min. Platelets were sedimented by centrifugation of the PRP at 800 × g for 15 min and washed with HEPES buffer (137 mM NaCl, 2.7 mM KCl, 1 mM MgCl_2_, 5.6 mM glucose, and 3.8 mM HEPES, pH 6.5) containing 0.35% BSA and 0.4 mM EGTA. The washed platelets were suspended in HEPES buffer (pH 7.4) and adjusted to 4 × 10^8^ cells/ml. Platelet aggregation was measured with an aggregometer (Chrono-Log Co.) according to Born’s turbidimetry method [21]. Briefly, washed platelet suspension was incubated at 37°C in the aggregometer with stirring at 1200 rpm, and then various concentrations of SH were added. After 3 min preincubation, platelet aggregation was induced by the addition of collagen (3 μg/ml), AA (100 μM), or thrombin (0.05 U/ml).

### Cell viability

Cell viability of platelets was determined as previously described [22]. Cell death of platelets by SH treatment was detection using a Cell Counting Kit-8 according to the manufacturer’s instructions (Wako, Osaka, Japan). *In vitro* viability was determined by measuring reduced formazan, a colorimetric assay based on the reduction of tetrazolium salt by cellular NADH or NADPH. The working solution (10 μl) containing WST-1 and SH was added to the PRP (200 μl) containing 4 × 10^8^ platelets/ml in a 96-well microtiter plate (Disposable Products, Adelaide, South Australia). The absorbance of the colored product (formazan dye) was read on a microplate reader (Well Reader SK601; Seikagaku, Tokyo, Japan) using a test wavelength of 450 nm against a reference wavelength of 650 nm.

### Serotonin secretion

Serotonin release was measured as previously described [20]. In brief, to prevent the reuptake of secreted serotonin, imipramine (a serotonin reuptake inhibitor, 5 μM) was added to PRP. Washed rabbit platelets were treated with various concentrations of SH at 37°C for 3 min prior to the addition of an agonist (collagen 3 μg/ml, AA 100 μM, or thrombin 0.05 U/ml) for 5 min. An aliquot (0.35 ml) of the washed rabbit platelets was mixed with 5 mM EDTA on ice and centrifuged at 12,000 × g for 2 min. The supernatant was mixed with 6 M trichloroacetic acid (TCA) and centrifuged at 12,000 × *g* for 2 min. An aliquot (0.3 ml) of the TCA supernatant was mixed with 1.2 ml of the solution (0.5% *o*-phthalaldehyde in ethanol diluted 1:10 with 8 N HCl), placed in a boiling water bath for 10 min, and then cooled on ice. The excess lipids were extracted with chloroform, and the fluorophore was measured at excitation and emission wavelengths of 360 nm and 475 nm, respectively. Serotonin creatinine sulfate was used as the standard solution to calculate the extent of serotonin release.

### Thromboxane B_2_ formation

Platelets were preincubated with SH or ASA at the indicated concentrations for 3 min and then exposed to collagen (3 μg/ml), AA (100 μM), or thrombin (0.05 U/ml), as in the aggregation assay. Ethylene glycol bis(2-aminoethyl ether) tetraacetic acid (EGTA, 2 mM) containing 0.1 M KCl and indomethacin (5 mM) were then added to platelet suspension. Thromboxane B_2_ (TXB_2_) level was measured with an enzyme-linked immunosorbent assay (ELISA) kit according to the manufacturer's instructions.

### Statistical analysis

Results are expressed as means ± SEM, and were analyzed using Student’s *t-*test or an analysis of variance (ANOVA). The results were considered significant when *P* < 0.05.

## Results

### Effect of SH on thrombus formation

**Figure 1:**
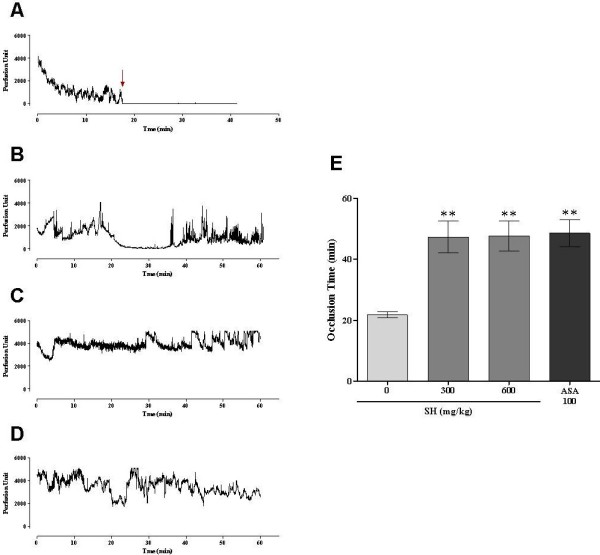
**Effects of SH on thrombus formation *in vivo*.** (**A**) Control, (**B**) SH 300 mg/kg, (**C**) SH 600 mg/kg, (**D**) ASA (aspirin) 100 mg/kg, and (**E**) bar graph is expressed from a representative data of eight individuals. Arrows show the occlusion point by thrombus formation. **P < 0.01 vs. 0 mg/kg.

To investigate the effects of SH on arterial thrombus formation *in vivo*, we used a rat carotid artery injury model induced by FeCl_3_. After 50% FeCl_3_ application, injured vessels of the control group were occluded within 21.8 ± 1.0 min. After oral SH treatment for 5 days, the time to form an occlusion was significantly longer, 25.5 ± 6.2 min and 25.9 ± 5.8 min at 300 mg/kg and 600 mg/kg of SH, respectively. As a positive control, ASA treatment for 5 days also prolonged occlusion time to 26.8 ± 5.4 min at 100 mg/kg (Figure 1). Taken together, SH showed an equivalent effect to ASA, although SH treatment was at higher doses than ASA.

### Effect of SH on aggregation and coagulation times *ex vivo*

**Figure 2:**
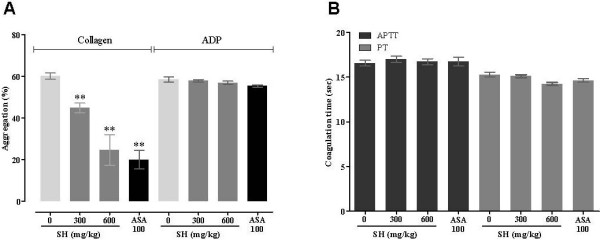
**Effects of SH on platelet aggregation and coagulation times *ex vivo*.** Platelet aggregation was induced by collagen (3 μg/ml) or ADP (5 μM) (**A**, n = 8). In the coagulation assay, PPP was obtained by centrifuging PRP, after which APTT and PT were measured as described (**B**, n = 9). **P < 0.01 vs. 0 mg/kg.

Figure 2A shows how SH inhibited collagen-induced aggregation in a concentration-dependent manner (25.3 ± 3.8% inhibition at 300 mg/kg and 59.0 ± 8.6% at 600 mg/kg). ASA also inhibited collagen-induced aggregation by 66.7 ± 5.9% at 100 mg/kg. However, SH treatment did not significantly change coagulation times, including APTT and PT. These data indicate that SH has excellent antiplatelet activity but does not affect coagulation.

### Effect of SH on washed rabbit platelet aggregation *in vitro*

**Figure 3:**
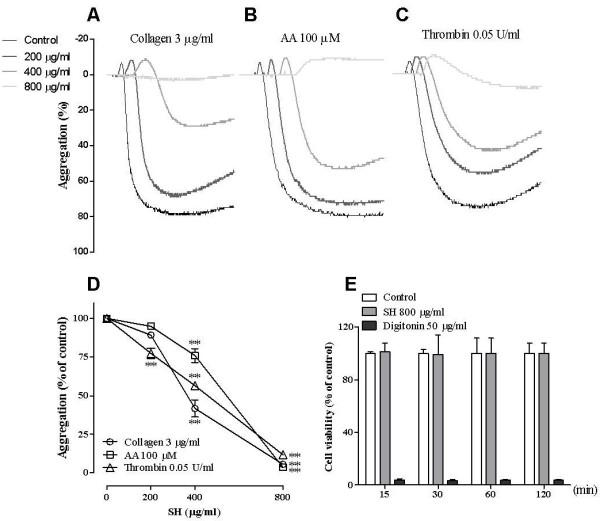
**Effects of SH on washed rabbit platelet aggregation and cell viability.** Aggregation is expressed as a percentage of maximum aggregation by the inducer. Cell viability was measured as described. Collagen (**A**), AA (**B**), thrombin (**C**), expressed as a graph from representative data (**D**, n = 4), and cell viability (**E**, n = 3). Data are expressed as the mean ± S.E.M. **P < 0.01 vs. 0 μg/ml.

To confirm the antiplatelet activity of SH, we investigated the effect of SH on various agonist-induced platelet aggregations. SH inhibited collagen- (Figure 3A), AA- (Figure 3B), and thrombin (Figure 3C)-induced rabbit platelet aggregations in a concentration-dependent manner (Figure 3D). In addition, a WST-1 assay also confirmed that the antiplatelet effect of SH was not due to cellular cytotoxicity (Figure 3E).

### Effect of SH on serotonin secretion

**Figure 4:**
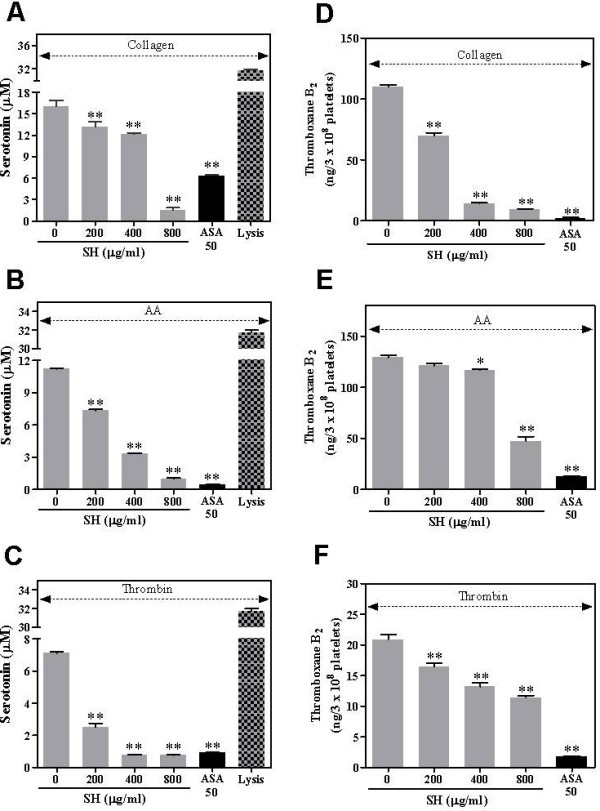
**Effects of SH on serotonin secretion and TXB**_**2 **_**formation.** Serotonin secretion was induced by (**A**) collagen, (**B**) AA, or (**C**) thrombin and was then determined by a fluorimetric method, as a described (n = 4). TXB_2_ formations were preincubated with SH or ASA at the indicated concentrations for 3 min and then exposed to (**D**) collagen (3 μg/ml), (**E**) AA (100 μM), or (**F**) thrombin (0.05 U/ml). Data are expressed as the mean ± SEM. **P < 0.01 vs. 0 μg/ml.

Serotonin is secreted from activated platelets during platelet aggregation [23]. Notably, SH inhibited serotonin secretion in a concentration-dependent manner, with inhibition percentages of 17.7%, 24.1%, and 90.1% for collagen (Figure 4A), 34.5%, 70.2%, and 91.1% for AA (Figure 4B), and 64.6%, 88.7%, and 89.0% for thrombin (Figure 4C) at 200, 400, and 800 μg/ml, respectively. ASA, as a positive control, potently inhibited serotonin secretion. In addition, total serotonin content of platelets was expressed as lysis (Figure 4).

### Effect of SH on thromboxane B_2_ formation

In the TXB_2_ formation assay, SH significantly inhibited collagen- (Figure 4D), AA- (Figure 4E), and thrombin (Figure 4F)-induced TXB_2_ formation. These results indicate that SH has an overall effect rather than a selective effect in platelet activation. In addition, ASA, a cyclooxygenase inhibitor, completely suppressed the production of TXB_2_ from AA by cyclooxygenase-1 activation.

## Discussion

In this study, we demonstrated two major findings: SH had an antithrombotic effect via antiplatelet activity, and the antiplatelet effect of SH involved the suppression of serotonin secretion and TXB_2_ production. These results suggests that SH may be used as an herbal formula to manage atherosclerosis and thrombotic disease, although it still needs further study with respect to its molecular mechanisms.

Activation and aggregation of platelets play an important role in thrombotic complications, such as atherosclerosis, stroke, myocardial infarction, and acute coronary syndromes [8-10]. In the clinical treatment for thrombotic diseases, inhibition of platelet activation leads to suppression of thrombosis formation and progression, and therefore, it is an important target for preventing complications after an acute coronary incident [24]. Generally, platelet aggregation and activation are mainly mediated through adhesion of platelets to the site of injury, and through the action of endogenous agonists such as collagen, ADP, and thrombin, followed by the release of TXA_2_ and serotonin, which act as amplification factors in platelet aggregation [25,26].

In this study, SH significantly prolonged the occlusion time of thrombus formation when applied in a FeCl_3_-induced thrombus formation model. Our results show that SH, at a concentration of up to 300 mg/kg, had an equivalent effect to ASA, although SH was administered at a higher dose than ASA (Figure 1).

SH inhibited collagen-induced platelet aggregation *ex vivo* (Figure 2A) in a concentration-dependent manner without affecting coagulation, including APTT and PT (Figure 2B), indicating that SH inhibits thrombus formation by antiplatelet activity rather than anticoagulant activity.

Accordingly, we investigated the effect of SH on various agonist-induced platelet aggregations to identify the antiplatelet activity. SH potently inhibited collagen-, AA-, and thrombin-induced platelet aggregation in a concentration-dependent manner (Figure 3) without cellular cytotoxicity (Figure 3E). In platelet activation, serotonin secretion is the indicator to identify the levels of platelet activation because serotonin is released from activated platelets during platelet aggregation [23]. SH significantly inhibited collagen-, AA-, and thrombin-induced serotonin secretion as well as agonist-induced TXB_2_ formation (Figure 4). TXA_2_, as the active form of TXB_2_, is the major contributor to platelet aggregation and activation [11]. Inhibition of serotonin and thromboxane B_2_ production in our results indicate that inhibition of platelets by SH may be an overall effect rather than a selective effect of platelet activation.

Additionally, the protective effects of *S. baicalensis* and *Z. officinale*, which are components of SH, have been reported in cardiovascular disease [17-19]. In a previous report, *S. baicalensis* prolonged thrombus formation by 23.79% due to its antithrombotic activity, and inhibited platelet aggregation by 45.52% due to its antiplatelet activity [17]. Also, *Z. officinale* a bioactive ginger, reportedly could have antiplatelet activity [18]. In comparison to previous results on the individual components of SH, our study suggests that SH has a complementary effect whereby all of the components work together to create an improved antithrombotic effect.

## Conclusions

Taken together, this study suggests that SH, which contains *S. baicalensis* and *Z. officinale*, inhibited thrombus formation through antiplatelet activity, resulting in the inhibition of platelet aggregation and suppression of serotonin and TXB_2_ production.

## Abbreviations

AA: Arachidonic acid; ACD: Anticoagulant citrate dextrose; ADP: Adenosine diphosphate; APTT: Activated partial thromboplastin time; ASA: Aspirin; BSA: Bovine serum albumin; DMSO: Dimethyl sulfoxide; EGTA: Ethylene glycol-bis(*β*-aminoethyl ether)-N,N,N',N'-tetraacetic acid; FVIIa: Factor VIIa; GP: Glycoprotein; OPT: *O*-phthalaldehyde; PRP: Platelet-rich plasma; PT: Prothrombin time; SH: Soshiho-tang; TCA: Trichloroacetic acid; TF: Tissue factor; TXA2: Thromboxane A_2_.

## Competing interests

The authors declare that they have no competing interests.

## Authors’ contributions

JJL, TK, YKC, and JYM participated in the design of the study; JJL and TK carried out the experiments, analyzed the data, and wrote the paper. All authors read and approved the final manuscript.

## Pre-publication history

The pre-publication history for this paper can be accessed here:

http://www.biomedcentral.com/1472-6882/13/137/prepub
